# Systematic analyses and comprehensive field synopsis of genetic association studies in hepatocellular carcinoma

**DOI:** 10.18632/oncotarget.9937

**Published:** 2016-06-10

**Authors:** Dong Dong, Yang Zou, Pan Zhang, Zhihong Wu

**Affiliations:** ^1^ Laboratory of Molecular Ecology and Evolution, Institute of Estuarine and Coastal Research, East China Normal University, Shanghai, 200062, P.R. China; ^2^ Beijing Key Laboratory for Genetic Research of Bone and Joint Disease, Beijing, 100730, P.R. China; ^3^ Central laboratory, Peking Union Medical College Hospital, Peking Union Medical College and Chinese Academy of Medical Sciences, Beijing, 100730, P.R. China

**Keywords:** hepatocellular carcinoma, meta-analyses

## Abstract

Hepatocellular carcinoma (HCC) is one of the most common malignancy in the world. In order to comprehensively examine the association between genetic variants and risk of HCC, a systematic literature search and meta-analyses of the evidences have been performed. With the data from 301 articles, we conducted meta-analyses for 69 polymorphisms involving 46 distinct genes. The result showed that 31 polymorphisms in 25 genes are significantly associated with HCC risk. Cumulative epidemiological evidence for a significant association with HCC risk was graded strong for one polymorphism (*NQO1* rs1800566). Furthermore, we provided a database to integrate and analyze the association of genetic variants and HCC risk. To the best of our knowledge, this is the first comprehensive field synopsis and systematic meta-analysis of genetic association with HCC risk. We have provided a useful resource and platform for investigators to explore the association of sequence polymorphisms and HCC risk.

## INTRODUCTION

Hepatocellular carcinoma (HCC) is the most common malignancy primary cancer and the third-leading cause of cancer mortality worldwide [1]. The prevalence of this cancer shows remarkable geographic heterogeneity, with the highest rates being observed among East Asian and African populations [1, 2]. Recently, the number of cases increased dramatically in Western countries, and it has been estimated that the annual number of new cases exceeds 700,000 worldwide [3]. Despite the rapid progress in diagnostic and therapeutic modalities, the overall 5-year survival rate for HCC is extremely low (18%) [4]. Many etiological factors for HCC has been reported [5]. Nearly 80% of the HCC are associated with infections with hepatitis B virus or hepatitis C virus. Alcoholic liver diseases and non-alcoholic fatty liver diseases are also major risk factors for HCC [6]. HCC is the combined result of a multi-stage, multi factor of long-term exposure and accumulation [7–10]. Multiple studies have revealed that cancer is a genetic disease, and is thought to develop through the acquisition of genetic alterations [11–13]. Many efforts have been devoted to uncover genetic aberrations in HCC [8, 14–16], such as point mutation in *p53* (TP53) and β*–catenin* (CTNNB1), etc. However, our understanding of genetic landscape in HCC is still far from complete and the key drivers of HCC tumourigenesis remain poorly to be understood.

Up to date, numerous works enrolling tens of thousands of subjects have been performed to examine the role of genetic variations in HCC carcinogenesis in the past two decades. Many sequence polymorphisms have been identified as potential genetic factors associated with HCC susceptibility. Notably, many studies might investigate the association between a specific polymorphism with HCC risk, however, the results of these works are not always consistent. Systematic review covering all tested polymorphisms is necessary. Here, we comprehensively evaluate the candidate-gene association studies of HCC risk, and perform meta-analyses for variants with sufficient data. We provided a systematic synopsis of our current understanding of the genetic basis of HCC susceptibility.

## RESULTS

### Characteristics of the eligible studies

**Figure 1:**
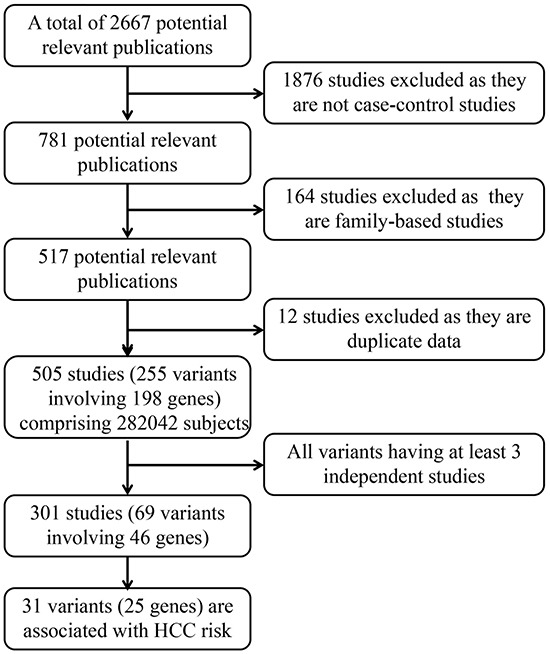
Profiles of literature search, meta-analysis and evaluation of cumulative evidence

In this work, we totally identified 505 eligible articles, comprising 282,042 subjects (case: 124,452, 44.1%). A total of 255 polymorphisms in 198 genes were eligible in our analysis (Figure 1). Most of these works (n=496, 98.22%) have been published since 2000. We conducted meta-analyses for 69 polymorphisms in 46 genes that had at least three data sources (301 eligible articles left). For the 69 main meta-analysis works, the mean sample size was 4087 (range from 607 to 14425) with an average of 7.6 independent studies.

### Meta-analyses

Detailed meta-analysis results were recorded for each of the polymorphisms. At first, we evaluated these polymorphisms using additive model. Among all the allele contrast meta-analysis, 21 (30%) polymorphisms in 19 genes showed nominally significant associations with HCC risk (*P-value* < 0.05). The number of subjects enrolled in the meta-analyses ranged from 607 to 14425 (mean: 4087, Table 1). The genotype distributions of these polymorphisms in the control group were all in accordance with the Hardy-Weinberg equilibrium (HWE). Strong associations with HCC (ORs > 2) have been detected for only one polymorphism (*PNPLA3* rs738409, OR=2.01). Moderate associations with HCC (ORs 1.5-2.0 0.5-0.8) were identified for 13 polymorphisms (Table 1). Five additional variants were significantly associated with HCC risk in meta-analyses stratified by ethnicity (Table 1). Three polymorphisms (*IL8* rs4073, OR=1.22; *COX-2* rs20417, OR=1.94; *MTHFR* rs1801133 OR=1.09) had associations with HCC risk only in Asian people. One polymorphism (*HFE* rs1799945, OR=1.73) only showed significantly association with HCC risk among African people, and one polymorphism (*mEPHX* rs1051740, OR=1.46) has the association with HCC risk only in Caucasian. Next, we performed ethnicity-specific analyses using either dominant or recessive genetic models to further evaluate the associations of genetic variants with HCC risk. Another 5 significant associated variants in 5 genes were identified using either dominant or recessive models (Table 2). Hepatitis B virus (HBV) infection is particular important risk factor for HCC. Many works have documented the association between genetic polymorphisms and risk of HBV-related HCC. Here, we explored these associations of 8 polymorphisms (Supplementary Table S1). The result showed that two polymorphisms are significantly correlated with HBV-related HCC (*TNF-α* rs1800630, OR=1.76; *IL28B* rs12979860 OR=1.70).

**Table 1:** Genetic variants nominally significantly associated with HCC risk in meta-analyses using additive model

Genes	Variants	Comparisons	Frequency (%)	Ethnicity	Number assessed			Hepatocellular carcinoma risk			Venice criteria grade	Cumulative evidence of association
					Studies	Cases	Controls	OR(95%CI)	P	Ph/I^2^		
Associations identified by analysis of all available data												
IL-1β	rs1143627	T vs C	0.47	All ancestries	8	2211	1854	0.83(0.72-0.96)	0.01	0.062/47.9%	ABA	Moderate
miR-196	rs11614913	T vs C	0.47	All ancestries	12	5703	6580	1.13(1.04-1.23)	0.004	0.005/59.2%	ACC	Weak
IL28B	rs12979860	C vs T	0.27	All ancestries	9	2803	1653	1.22(1.00-1.49)	0.043	0.006/62.8%	BCC	Weak
XPD	rs1799793	G vs A	0.16	Asian	5	2005	2164	1.26(1.03-1.55)	0.024	0.066/54.6%	BCC	Weak
TGF-β1	rs1800469	C vs T	0.46	All ancestries	11	3180	4424	1.18(1.01-1.37)	0.034	0/76.1%	ACC	Weak
HFE	rs1800562	G vs A	0.05	All ancestries	11	1225	3858	1.44(1.02-2.03)	0.039	0.03/51.2%	ACC	Weak
NQO1	rs1800566	C vs T	0.41	All ancestries	3	745	1091	1.34(1.17-1.54)	0	0.404/0%	AAA	Strong
TNF-α	rs1800629	G vs A	0.10	All ancestries	20	3414	3979	1.45(1.09-1.94)	0.012	0.000/80.0%	BCC	Weak
TNF-α	rs1800630	C vs A	0.18	All ancestries	7	1679	1833	1.39(1.10-1.74)	0.005	0.023/59.2%	BCB	Weak
IL6	rs1800795	G vs C	0.21	All ancestries	3	219	522	0.72(0.53-0.98)	0.039	0.993/0%	BAA	Moderate
IL10	rs1800872	A vs C	0.44	Asian	6	1540	2173	1.13(1.02-1.26)	0.023	0.387/4.6%	BAC	Weak
IL8	rs2227306	C vs T	0.36	Asian	3	486	698	0.79(0.62-1.00)	0.045	0.176/42.4%	BBA	Moderate
MDM2	rs2279744	T vs G	0.47	All ancestries	11	2905	3495	0.73(0.61-0.88)	0.001	0.000/78.4%	ACA	Weak
CTLA4	rs231775	G vs A	0.34	Asian	3	1369	1426	1.34(1.03-1.75)	0.029	0.011/77.7%	BCB	Weak
XRCC1	rs25487	A vs G	0.41	All ancestries	23	4851	5508	1.24(1.10,1.39)	0	0.000/66.0%	ACB	Weak
HLA-DQ	rs2856718	A vs G	0.44	Asian	3	2756	3681	0.73(0.67-0.78)	0	0.395/0%	BAA	Moderate
miR-146a	rs2910164	C vs G	0.42	All ancestries	16	6179	8246	1.10(1.03-1.18)	0.004	0.067/37.2%	ABC	Weak
TNF-α	rs361525	G vs A	0.05	All ancestries	12	1529	1836	1.44(1.04-2.00)	0.027	0.102/36.1%	ABA	Moderate
EGF	rs4444903	G vs A	0.40	All ancestries	12	2304	3664	0.83(0.74-0.94)	0.003	0.037/46.8%	ABA	Moderate
PNPLA3	rs738409	C vs G	0.33	Caucasian	9	1234	2624	2.01(1.56-2.35)	0	0.000/74.3%	BCB	Weak
BIRC5	rs9904341	G vs C	0.46	All ancestries	3	473	933	0.84(0.71-0.99)	0.032	0.886/0%	BAA	Moderate
Associations identified from additional analyses by ethnic group												
IL8	rs4073	A vs T	0.49	Asian	3	691	906	1.22(1.06-1.42)	0.007	0.583/0.0%	BAA	Moderate
Cox-2	rs20417	G vs C	0.07	Asian	4	1168	1438	1.94(1.03-3.67)	0.041	0.393/0.0%	BAA	Moderate
EPHX1	rs1051740	C vs T	0.30	Caucasian	3	249	495	1.46(1.14-1.86)	0.002	0.393/0.0%	BAA	Moderate
HFE	rs1799945	C vs G	0.17	African	3	235	362	1.73(1.27-2.37)	0.001	0.567/0.0%	BAA	Moderate
MTHFR	rs1801133	C vs T	0.41	Asian	7	2427	3449	1.09(1.01-1.18)	0.038	0.713/0.0%	AAC	Weak

**Table 2:** Genetic variants nominally significantly associated with HCC risk in meta-analyses using dominant and recessive model

Genes	Variants	Comparisons	Frequency	Ethnicity	Number assessed			Allelic contrasts			Hepatocellular carcinoma risk				Venice criteria grade	Cumulative evidence of association
					Studies	Cases	controls	OR(95%CI)	P	Ph/I^2^	Model	OR(95%CI)	P	Ph/I^2^		
OGG1	rs1052133	C vs G	0.407455	Asian	9	2424	2271	1.21(0.92-1.59)	0.182	0.000/88.8%	REC	1.34(1.02-1.77)	0.039	0.000/72.5%	ACC	weak
MTHFR	rs1801131	A vs C	0.400447	ALL	6	2030	3096	0.96(0.87-1.06)	0.42	0.783/0.0%	DOM	0.66(0.45-0.98)	0.038	0.219/27.4%	ABA	Moderate
XRCC3	rs861539	C vs T	0.145481	Asian	7	2331	2759	1.21(0.92-1.59)	0.182	0.000/88.8%	DOM	2.89(1.57-5.31)	0.001	0.014/62.5%	ACC	weak
GSTP1	rs1695	A vs G	0.47543	Asian	4	902	1051	1.08(0.82-1.42)	0.6	0.054/60.7%	DOM	1.65(1.18-2.32)	0.004	0.559/0.0%	BAA	Moderate
IL-1β	rs16944	C vs T	0.478646	Asian	6	998	1037	0.88(0.68-1.15)	0.359	0.009/67.3%	REC	0.74(0.56-0.99)	0.044	0.221/28.6%	ABA	Moderate

To assess the cumulative epidemiologic evidence for significant meta-analysis, Venice criteria [17] were applied. Epidemiological credibility were scored as ‘strong’, ‘moderate’ or ‘weak’ by a composite assessment, including the amount of evidence, extent of replication, and protection from bias. For the amount of evidences, 14 grades of ‘A’, 16 grades of ‘B’, and 0 grades of ‘C’ were given. For the extent of replication, 10 grades of ‘A’, 7 grades of ‘B’, and 13 grades of ‘C’ were given. For the protection from bias, 16 grades of ‘A’, 4 grades of ‘B’, and 10 grades of ‘C’ were given. One polymorphism (*NQO1* rs1800566) was graded strong for evidence of association with HCC risk using Venice criteria result. Moreover, moderate and weak for the evidence of true association with HCC were assigned to 14 and 16 polymorphisms, respectively.

Previous meta-analyses works have independently analyzed the association between genetic variants and risk of HCC [18–22]. Here, we comprehensively compiled these works, and compared the differences with our results (Supplementary Table S2). The result showed that a total of 42 polymorphisms have been reported by previous meta-analyses, and the results of seven polymorphisms (rs11614913, rs1143627, rs25487, rs2910164, rs1801131, rs17401966 and rs861539) are inconsistent with our work because of their limited data resources. A total of 27 polymorphisms have been comprehensively evaluated for the first time.

### HCCdb: a database of HCC-related polymorphisms

To facilitate HCC related polymorphisms integration and online query, we subsequently constructed a database of HCC-related polymorphisms (HCCdb). Currently, HCCdb contains 69 polymorphisms in 46 genes. The current version of HCCdb provides a user friendly search engine, which allows to search the basic content of gene or literature information. Furthermore, HCCdb provided a module to carry out a direct meta-analysis on the polymorphisms. Users can select the genetic model and effect models when performing online meta-analysis. In HCCdb, the OR and 95% CI can be measured to evaluate the strength of associations between polymorphisms and HCC risk. The database is freely available at http://donglab.ecnu.edu.cn/databases/HCCdb/.

## DISCUSSION

Sequence polymorphisms in genes have been considered as underlying candidates in hepatocellular carcinogenesis. In this work, we described the results of the first systematic synopsis and meta-analysis in the field of genetic predisposition to HCC. We found that 31 polymorphisms in 25 genes showed significant associations with HCC risk. Our work provided a comprehensive research synopsis of candidate-gene association study of HCC risk. Using Venice criteria results, we graded one polymorphism strong for cumulative epidemiological evidence of association with HCC risk (*NQO1* rs1800566). *NQO1* is a cytosolic enzyme and plays an important role in protecting cells against oxidative stress by catalyzing two-electron reduction of numerous quinoid compounds into their less toxic form. Many epidemiological studies have investigated the effect of *NQO1* rs1800566 polymorphism on carcinogenesis [23, 24], and the effect seems diverse in different malignant tumors. This work showed that the *NQO1* rs1800566 polymorphism is a critical risk factor for HCC risk (OR=1.34). However, we failed to evaluate the susceptibility of *NQO1* rs1800566 polymorphism to HCC in specific populations because of the limited eligible published case–control studies.

There are still several deficiencies in this work. First, although we have thoroughly searched the literature in PubMed database to identify eligible studies, it is possible that some studies might have been missed. To extend our search, we also checked the related meta-analysis in Google Scholar linking multiple databases. Second, we did not evaluate gene-gene interactions or gene-environment interactions. More additional studies specifically designed to detect these interactions are needed. Third, although Venice criteria offer the advantage for assessing various sources of potential bias, some of the indicators are difficult to measure, such as genotyping error, population stratification and phenotype misclassification.

To the best of our knowledge, this work is the largest and most comprehensive assessment of the literature on the genetic association with HCC susceptibility conducted to date. This work not only summarizes the current literature linking to genetic epidemiology of HCC, but also gives comprehensive data and helpful clues for designing future studies to further investigate genetic risk factors for HCC. In the future, the updating of web-based data collection for disease related studies would help to improve the cumulative evidence for genetic associations in HCC.

## MATERIALS AND METHODS

### Literature search and selection criteria

To identify the genetic variants associated with HCC risk, we searched the PubMed database by using the following keywords: “(liver cancer OR hepatocellular carcinoma) AND (polymorphism OR polymorphisms)” in title field without language restrictions for studies published up to 18 June 2015. Furthermore, Google scholar was also used to search eligible publications using the same keywords. This search produced 2667 potentially relevant publications. After further evaluation, 505 eligible publications were retained (Figure 1). All the eligible studies should met the following criteria: 1) Publications must be published in a peer-reviewed journal; 2) the study used a case-control and other appropriate cohort design in human beings were included; 3) Family-based studies were excluded; 4) HCC cases were diagnosed by pathological and/or histological examination, excluding liver cirrhosis, chronic hepatitis B, acute liver failure, asymptomatic HBV carriers and so on; 5) sufficient genotype data were presented to calculate the odd ratios (ORs) and 95% confidence intervals (CIs).

### Data extraction

Data were independently extracted by two reviewer (PZ and YZ) and then checked by another reviewer (DD). The results of a total of 16 publications have some inconsistent and disagreements. All of these disagreements were encountered because of careless. We collected the disagreements and discuss with DD, and made the final decision after re-performing the work. The study provided enough information for the genotypic or allelic distribution of individual variants for both HCC cases and controls was the one we needed. Following characteristics were collected: first author's surname, publication year, ethnicity (categorized as Caucasian, Asian, African, or mixed, including more than one ethnic category [25]), dbSNP ID, gene symbol, variants, source of control (hospital based, population based, family based), numbers of cases and controls of different genotypes, genotyping method, PubMed ID, and the type of the infected virus, respectively.

### Statistical analysis

For the stabilization of heterogeneity test statistic (*I*^2^) and the operation of sensitivity analyses, we performed meta-analyses for the 69 genetic variants with case-control data available containing at least three independent sources. All statistical tests were conducted by STATA, version 12.0. All tests were two-tailed, and only *P-value*<0.05 was considered significant. To comprehensively analyze the relationship between genetic variants and risk of HCC, we selected three genetic models: additive model, dominant model and recessive model. To illustrate the models, we assume a polymorphism genome locus having two alleles, labeled A and a. A is the high-risk candidate allele and a is the lower-risk allele. Additive model is the same as allele model, represent the effect of the A allele vs. the a allele; dominant model represent the effect of the a/a+A/a vs. the A/A genotypes only when present in two copies of A allele, recessive mode represent the effect of the A/A+A/a vs. the a/a genotypes when present in either one or two copies of A allele. Summary odds ratios (ORs) with 95% confidence intervals (CIs) for alleles or genotypes were used to assess strength of associations between genetic variants and HCC risk by the random-effects method [26]. In the primary analyses, pooled ORs were acquired for allele contrast. In addition, dominant and recessive models were also assessed on all eligible polymorphisms. For some specific variants, like GSTM1 and GSTT1 ‘Present/Null’, conventional comparisons were used in original studies. If data permitted (at least 3 data source), we also performed subgroup analyses by ethnicities. For common variants (MAF≥5%), the minor allele and major allele sometimes were reversed in different ethnicities, it may lead to deviation. To minimize false-negative errors, for variants that showed no evidence of association with HCC risk in the meta-analyses, only those with admission of six independent datasets were selected for presentation.

Heterogeneity assumption was estimated by Chi-square based on Q-test [27]. *I*^2^ statistic was also used to assess heterogeneity [28]. Generally, *I*^2^ values less than 25% correspond to mild heterogeneity, values between 25% and 50% correspond to moderate heterogeneity, and values greater than 50% correspond to large heterogeneity between studies. Sensitivity analyses was performed excluding studies whose allele frequencies in controls exhibited significant deviation from the Hardy-Weinberg Equilibrium (HWE), given that the deviation may denote bias. Moreover, the extent to which the combined risk estimate might be influenced by individual studies was assessed consecutively omitting every study from the meta-analyses (leave-one-out sensitivity analyses). Begg's funnel plots [29] and Egger's linear regression test [30] were used to investigate the publication bias.

### Evaluation of cumulative evidence

In order to assess statistically significant associations identified by meta-analyses, Venice criteria was employed in this work. It grades the cumulative evidence at three major criteria: (i) amount of evidence; (ii) replication of results; (iii) publication from bias. Amount of evidence was graded by the sum of test alleles or genotypes among both cases and controls in the meta-analysis; ‘A’ for over 1,000, ‘B’ for 100 to 1,000, and ‘C’ for less than 100. Caution was taken when applying this criterion to rare variants with frequency <1%, as an A grade is unobtainable. Replication was graded by the heterogeneity statistic; ‘A’ for *I*^2^ < 25%, ‘B’ for *I*^2^ between 25% and 50%, and ‘C’ for *I*^2^ >50%. Protection from bias may be caused by factors that lead to systematic deviations from the true effect of a genetic association. Biases may operate at the level of a single study, a collection of studies (e.g. meta-analysis), or a research field at large. It can be graded as ‘A’ if there was no observable bias and bias was unlikely to explain the presence of the association, ‘B’ if bias could be present or could explain the presence of the association, or ‘C’ if bias was evident or was likely to explain the presence of the association. Assessment of protection from bias also included consideration of the magnitude of the association; a score of ‘C’ was assigned to an association with a summary OR < 1.15 unless the association had been replicated prospectively by multiple studies with no evidence of publication bias. Cumulative epidemiological evidence was defined as strong, moderate, or weak. If all three grades were A, we considered it was strong. While if one or more grades were C, it was weak. All other combinations was moderate.

### Database construction

The HCCdb system is based on a three-tier architecture: client, server and database. It includes a user-friendly web interface, PHP's DBI module and MySQL database. HCCdb was developed on MySQL v4.1 with the MyISAM storage engine.

## SUPPLEMENTARY TABLES


