# Supplementary material for: Systematic analyses and comprehensive field synopsis of genetic association studies in hepatocellular carcinoma
Source: Oncotarget. 2016 Jun 10;7(29):45757–63. doi: 10.18632/oncotarget.9937 (PMC5216758; doi:10.18632/oncotarget.9937)
Supplement: Supplementary file 1 [file oncotarget-07-45757-s001.pdf]

Systematic analyses and comprehensive field synopsis of genetic association studies in hepatocellular carcinoma

SUPPLEMENTARY TABLES

Supplementary Table S1: Genetic variants nominally significantly associated with HBV-related HCC risk in meta-analyses

	HBV	Overall
	**OR(95%CI)**	**OR(95%CI)**
rs4680	0.88(0.64,1.20)	0.95(0.78,1.16)
rs16944	0.87(0.68,1.10)	0.88(0.72,1.07)
rs1143627	0.90(0.77,1.05)	**0.83(0.72,0.96)**
rs1799724	0.91(0.66,1.25)	1.13(0.82,1.57)
rs1800630	**1.76(1.13,2.74)**	**1.39(1.10,1.74)**
rs4646903	1.07(0.89,1.27)	0.99(0.80,1.24)
rs8099917	1.41(0.98,2.03)	1.40(0.80,2.44)
rs12979860	**1.70(1.02,2.82)**	**1.22(1.00,1.49)**

Supplementary Table S2: Comparing our results and previous meta-analysis works

Polymorphisms	This work		Previous works		Results
	**OR(95%)**	**Data sources**	**PubMed ID**	**Data sources**	
rs2279744	1.362(1.132,1.638)	11	21565629	5	Consistent
rs11614913	1.133(1.041,1.234)	12	23691458	4	Inconsistent
rs62559044	1.002(0.745,1.348)	3	21798758	3	Consistent
rs1801133	1.090(0.963,1.233)	13	19930673	10	Consistent
rs1800630	1.385(1.104,1.737)	7	21336601	5	Consistent
rs1799964	1.132(0.878,1.458)	3	21336601	3	Consistent
rs1799724	1.130(0.815,1.567)	4	21336601	3	Consistent
rs12979860	1.220(0.997,1.493)	9	24085431	5	Consistent
rs1800469	1.176(1.012,1.367)	11	22257092	5	Consistent
rs1800470	0.998(0.753,1.323)	8	22257092	6	Consistent
rs738409	1.914(1.557,2.352)	9	24114809	7	Consistent
rs1143627	0.827(0.716,0.956)	8	21107607	5	Inconsistent
rs16944	0.877(0.719,1.070)	8	21107607	5	Consistent
rs1800562	1.438(1.018,2.031)	11	20196837	9	Consistent
rs1799945	1.141(0.897,1.453)	11	20196837	8	Consistent
rs1800629	1.451(1.085,1.940)	20	20819413	10	Consistent

(*Continued*)

Polymorphisms	This work		Previous works		Results
	OR(95%)	Data sources	PubMed ID	Data sources	
rs1042522	1.060(0.969,1.158)	17	22613405	7	Consistent
rs25487	1.240(1.104,1.393)	23	21645210	11	Inconsistent
rs1048943	1.122(0.827,1.521)	8	22297691	8	Consistent
rs4646903	0.993(0.797,1.237)	8	22297691	8	Consistent
rs4444903	0.830(0.736,0.937)	12	22403631	8	Consistent
rs2910164	1.103(1.032,1.180)	16	22768213	5	Inconsistent
rs4938723	1.151(0.974,1.360)	3	23935875	3	Consistent
rs3746444	1.212(0.957,1.535)	11	23155448	2	Consistent
rs1801131	0.959(0.865,1.062)	7	23457501	6	Inconsistent
rs17401966	0.894(0.680,1.175)	5	23634229	5	Inconsistent
rs361525	1.442(1.042,1.995)	12	20953524	6	Consistent
rs1800566	1.340(1.165,1.542)	3	24532470	3	Consistent
rs1800795	0.724(0.532,0.984)	3	24318992	2	Consistent
rs861539	1.470(0.986,2.190)	7	23558966	5	Inconsistent
rs2234922	0.742(0.528,1.043)	8	23955801	8	Consistent
rs689466	0.882(0.759,1.025)	7	23494177	5	Consistent
rs2031920	0.844(0.686,1.040)	21	22249978	15	Consistent
rs25489	1.174(0.962,1.433)	11	23055199	5	Consistent
rs1052133	1.207(0.915,1.593)	9	23271362	6	Consistent
rs13181	1.295(0.880,1.904)	9	23271362	7	Consistent
rs1799782	1.066(0.882,1.287)	9	23055199	5	Consistent
rs1800796	0.978(0.878,1.089)	7	24318992	3	Consistent
rs1051740	0.953(0.731,1.244)	12	23955801	12	Consistent
rs1800872	1.133(1.017,1.262)	6	22025883	4	Consistent
rs1800896	1.355(0.851,2.156)	4	22025883	4	Consistent
rs1800871	0.989(0.860,1.136)	4	22025883	3	Consistent
